# Cytotoxicity and cell cycle arrest induced by andrographolide lead to programmed cell death of MDA-MB-231 breast cancer cell line

**DOI:** 10.1186/s12929-016-0257-0

**Published:** 2016-04-16

**Authors:** Malabika Banerjee, Subrata Chattopadhyay, Tathagata Choudhuri, Rammohan Bera, Sanjay Kumar, Biswajit Chakraborty, Samir Kumar Mukherjee

**Affiliations:** Department of Microbiology, University of Kalyani, Kalyani, 741235 WB India; TCG Life Science Ltd., Bengal Intelligent Park, Tower-B, Block-EP & GP, Sector-5, Salt Lake, Kolkata, 700091 India; Department of Biotechnology, Visva-Bharati, Santiniketan, 731235 India

**Keywords:** Andrographolide, Apoptosis, Breast cancer, Phytomedicine, Cell cycle arrest, Bioanalysis

## Abstract

**Background:**

Breast cancer is considered as an increasing major life-threatening concern among the malignancies encountered globally in females. Traditional therapy is far from satisfactory due to drug resistance and various side effects, thus a search for complementary/alternative medicines from natural sources with lesser side effects is being emphasized. *Andrographis paniculata*, an oriental, traditional medicinal herb commonly available in Asian countries, has a long history of treating a variety of diseases, such as respiratory infection, fever, bacterial dysentery, diarrhea, inflammation etc. Extracts of this plant showed a wide spectrum of therapeutic effects, such as anti-bacterial, anti-malarial, anti-viral and anti-carcinogenic properties. Andrographolide, a diterpenoid lactone, is the major active component of this plant. This study reports on andrographolide induced apoptosis and its possible mechanism in highly proliferative, invasive breast cancer cells, MDA-MB-231 lacking a functional p53 and estrogen receptor (ER). Furthermore, the pharmacokinetic properties of andrographolide have also been studied in mice following intravenous and oral administration.

**Results:**

Andrographolide showed a time- and concentration- dependent inhibitory effect on MDA-MB-231 breast cancer cell proliferation, but the treatment did not affect normal breast epithelial cells, MCF-10A (>80 %). The number of cells in S as well as G_2_/M phase was increased after 36 h of treatment. Elevated reactive oxygen species (ROS) production with concomitant decrease in Mitochondrial Membrane Potential (MMP) and externalization of phosphatidyl serine were observed. Flow cytometry with Annexin V revealed that the population of apoptotic cells increased with prolonged exposure to andrographolide. Activation of caspase-3 and caspase-9 were also noted. Bax and Apaf-1 expression were notably increased with decreased Bcl-2 and Bcl-xL expression in andrographolide-treated cells. Pharmacokinetic study with andrographolide showed the bioavailability of 9.27 ± 1.69 % with a C_max_, of 0.73 ± 0.17 μmol/L and T_max_ of 0.42 ± 0.14 h following oral administration. AG showed rapid clearance and moderate terminal half lives (T_1/2_) of 1.86 ± 0.21 and 3.30 ± 0.35 h following IV and oral administration respectively.

**Conclusion:**

This investigation indicates that andrographolide might be useful as a possible chemopreventive/chemotherapeutic agent for human breast cancers.

**Electronic supplementary material:**

The online version of this article (doi:10.1186/s12929-016-0257-0) contains supplementary material, which is available to authorized users.

## Background

The plant, *Andrographis paniculata* Nees (family: Acanthaceae) is very common in Asian countries including India and has been reported to have a traditional therapeutic use [1, 2]. This plant is considered as an important source of phytomedicine to treat a wide range of diseases, such as respiratory infection, fever, bacterial dysentery and diarrhea [3, 4]. It was also reported to possess anti-inflammatory [5], antimalarial [6], and even anti-HIV activity [7]. The major bioactive component extracted from *A. paniculata* Ness is andrographolide, a diterpene lactone. Three hydroxyls at C-3 (secondary), C-14 (allylic) and C-19 (primary) on the basic structural skeleton were reported to be responsible for its biological activities [8, 9]. In recent years several studies have indicated that andrographolide also possess antitumor activity [10, 11].

Breast cancer is a major life-threatening concern among the malignancies encountered in females and ranks second as a cause of death [12]. Apoptosis is a programmed cell death which occurs due to the activation of certain evolutionarily conserved intracellular functions. Many naturally occurring phytochemicals were reported to possess anti-tumor effect thus inducing apoptosis of cancer cells. Curcumin from turmeric, epigallocatechin gallete from green tea, resveratrol from grape seed extract and quercetin from fruits are some examples of chemopreventive agents derived from plant that induce apoptosis with some being in clinical intervention trials [13, 14]. Earlier reports based on the pharmacological properties of andrographolide, especially on its antitumorogenic activity through various mechanisms, such as, inhibiting cell cycle progression, reducing invasiveness of cancer cells or inducing apoptosis through different cell-death mechanism in different carcinoma cells [10, 15] prompted us to evaluate the possible induction of apoptosis by andrographolide on breast cancer cell line.

With this background, this study was designed to evaluate in vitro anticancer activity of andrographolide in a breast cancer cell line, MDA-MB-231 which is highly invasive, proliferative, estrogen receptor (ER) negative and harbors mutated p53. Although, earlier studies with other breast cancer cells containing functional ER and wild type p53 showed cell growth inhibition and apoptosis induced by andrographolide [16, 17], reports on the effect on this particular triple negative breast cancer (TNBC) cell line are scanty. Therefore, it is worthwhile to investigate the inhibitory and/or apoptosis inducing effect of andrographolide on MDA-MB-231 as this cell line is clinically harder to treat [18]. Cancer cells harboring mutated p53 is exhibited as more resistant to certain anticancer drugs because mutated p53 no longer renders the tumor suppressing abilities of the wild type, rather it often contributes to the oncogenic characteristics [19]. Furthermore, metastatis-derived MDA-MB-231 breast cancer cell line is not hormone sensitive (ER negative). Blocking the Estrogen receptor in these cells will not serve the purpose of inhibiting cancer. Thus MDA-MB-231 cells are more resistant to drug therapy in comparison to other breast cancer cells like MCF-7. For instance, while resveratrol inhibits cell proliferation and activity in both MCF-7 and MDA-MB-231 cells, it was able to induce apoptosis in MCF-7 cells only [20]. In the present study, attempts have been made to elucidate the molecular mechanism by which andrographolide renders its inhibitory effects on cell proliferation, cell cycle, expression levels of pro- and anti-apoptotic proteins and finally towards apoptosis in this clinically distinct cell line. Our results show that andrographolide can inhibit the cellular growth of MDA-MB-231 by causing cell cycle arrest and apoptosis in a time- and dose-dependent manner.

Additionally, andrographolide was analyzed by LC-MS/MS method to determine its pharmacokinetic characteristics in the plasma of BALB/c mice and these pharmacokinetic results are important for further study of the clinical applications of andrographolide.

## Methods

### Materials and reagents

Andrographolide was procured from Santa Cruz Biotechnology (Santa Cruz, CA, USA), dissolved in DMSO and kept at 4 °C at a concentration of 50 mM. AnnexinV-FITC Apoptosis Detection Kit was purchased from BD Pharmingen (Pharmingen, USA). Caspases fluoremetric assay kit was purchased from Chemicon International Corporation (USA). Ac-DEVD-CHO (caspase-3 inhibitor), and Ac-LEHD-CHO (caspase-9 inhibitor) were from Calbiochem (La Jolla, USA). Primary antibodies (Bcl-2, Bcl-xL, Bax, Apaf-1, cytochrome *c*, β-actin and COX IV) and polyclonal secondary antibody were obtained from Santa Cruz Biotechnology Inc. (Santa Cruz, USA). The fetal bovine serum (FBS), Dulbecco’s Modified Eagle’s Medium (DMEM), and antibiotics were purchased from Gibco BRL (Grand Island, USA). Plastic wares for cell culture were procured from NUNC (Roskilde, Denmark). Other chemicals including 4, 6-diamidino-2-phenylindole (DAPI), 3[4-dimethylthiazol-2-71]-2-5-diphenyl tetrazolium bromide (MTT) were from Sigma-Aldrich (St. Louis, USA).

### Cell culture

MDA-MB-231, T-47D and MCF-7 human breast cancer cell lines and MCF-10A normal human breast epithelial immortalized cell line were obtained from American Type Culture Collection (ATCC) (Rockville, USA). MDA-MB-231, T-47D and MCF -7 breast cancer cells were cultured in DMEM supplemented with 10 % (*v/v*) heat inactivated FBS, 2 mM L-glutamine, 100 U/ml of penicillin, and 100 μg/ml of streptomycin. MCF-10A cells were maintained in DMEM-F12 (Sigma-Aldrich, USA) supplemented with 10 % (*v/v*) FBS, 100 U/ml of penicillin, 100 μg/ml of streptomycin, 2 mM of L-glutamine, 20 ng/ml of epidermal growth factor (Sigma-Aldrich, USA), 10 μg/ml of insulin (Sigma-Aldrich, USA), 100 ng/ml of cholera toxin (Sigma-Aldrich, USA) and 1 μg/ml of hydrocortisone (Sigma-Aldrich, USA). Cells were cultured in 75 cm^2^ culture flasks at 37 °C under humid environment in an incubator having 5 % CO_2_.

### Animals

Female BALB/c mice were obtained from animal facility, TCGLS, Kolkata, India. Animal studies involving mice were approved by the Institutional Animal Ethics Committee (IAEC), TCGLS with respect to ethical practice and animal care under the guidelines of Committee for the Purpose of Control and Supervision of Experiments on Animals (CPCSEA), India and Reg. No. 1068/PO/RcBi/S/07/CPCSEA.

Experimental animals, aged 6–8 weeks and weighed between 19–22 g, were maintained at temperature of 22 ± 2 °C, 30–70 % humidity and 12/12 h light-dark cycle. The mice had *ad libitum* access to standard animal diet and water.

### In vitro cytotoxicity assay

The effect of andrographolide on cell viability was measured by MTT assay following the method by Mosmann [21]. Briefly, the cells (1 × 10^5^ cells per ml) were seeded in a 96 well micro titer plate (100 μl per well) with replications. Treatment was conducted for 24 and 48 h with different concentrations (0, 5, 7.5, 15, 30, 45, 60, 75 and 100 μM) of andrographolide. After incubation, 20 μl of 5 mg/ml MTT stock solution was added to each well and incubated for 4 h at 37 °C. The obtained formazan crystals were solubilized with DMSO and the absorbance was measured at 570 nm using a microplate reader (SpectraMax M5, Molecular Devices, USA). Cell viability (%) has been shown as a ratio of absorbance (A_570_) in treated cells to absorbance in control cells (0.1 % DMSO) (A_570_). The IC_50_ was calculated as the concentration of sample needed to reduce 50 % of the absorbance in comparison to the DMSO-treated control. Percent cell viability was calculated following the equation:

$$ \mathrm{Cell}\ \mathrm{viability}\left(\%\right) = \left[{\mathrm{A}}_{570}\left(\mathrm{Sample}\right)/{\mathrm{A}}_{570}\left(\mathrm{Control}\ \mathrm{DMSO}\right)\right]\times 100 $$

### Cell proliferation assay

MDA-MB-231 cells harvested from exponential growth phase were seeded at a density of 1 × 10^4^/well in a 24-well culture plate. After 24 h, the cells were treated with varying concentrations of andrographolide (15, 30, 45 and 60 μM) or DMSO (0.1 %, vehicle control) for 12, 24, 36 and 48 h. After treatment, cells were harvested by trypsinization, stained with 0.4 % trypan blue and counted in a hemocytometer. Cell proliferation index PI, was calculated as PI = C_t_/C_0_; where C_t_ = cell count at time t and C_0_ = cell count at the time of treatment.

### Analysis of apoptosis observing the nuclear and cell morphological changes

To study nuclear and chromatin structural changes, MDA-MB-231 cells were treated with or without 50 % inhibitory concentration (IC_50_) of andrographolide for 24 and 48 h respectively. The cell suspensions were harvested and the cell pellet was washed with DAPI-methanol (working solution, 1 μg/ml) twice. Cells were then incubated with 2 ml DAPI-methanol for 15 min at 25 °C. After centrifugation at 1000 × g, DAPI-methanol was discarded and the pellet was suspended in PBS, then mounted on a glass slide and 4 % paraformaldehyde was used as a fixative. Cells were examined using a fluorescence microscope (excitation, 360 nm; emission, 454 nm) (Nikon eclipse TE300, Tokyo, Japan) [22]. Cells with condensed chromatin and fragmented nuclei were considered as apoptotic.

Standard acridine orange/ethidium bromide (AO/EtBr) staining technique was used with some modifications to discriminate the live, apoptotic and necrotic cells [23]. Briefly, cells treated with or without 50 % inhibitory concentration (IC_50_) of andrographolide for 24 and 48 h, were stained with AO/EtBr (50 μg/ml each at 1:1), and then fixed on slide with 4 % paraformadehyde and analyzed under fluorescence microscope (excitation, 488 nm; emission, 550 nm).

### Cell cycle analysis

In order to study the stages of the cell cycle affected by andrographolide, flow cytometry analysis of cell cycle was carried out. Cells were seeded in a 6-well tissue culture plate at an initial density of 2.5 × 10^5^ cells per well in the presence of andrographolide (30 μM) or vehicle (DMSO) for 24, 36 and 48 h. The treatment medium and the attached cells were collected after trypsinisation and centrifugation at 1000 × g. Cells were then fixed with 70 % ethanol at 4 °C overnight. The resulted pellets were resuspended in PBS having 100 μg/ml RNAse A (Sigma-Aldrich, USA) and 50 μg/ml PI, and then kept in dark at 25 °C for 30 min. Cell cycle phase distribution were monitored on the BD FACSVerse™ (Becton Dickinson, USA) and analyzed by using FACSuite™ software. A total of 10,000 events were recorded.

### Externalization of phosphatidyl serine and confirmation of apoptosis

In order to examine phosphatidyl serine externalization from inner to the outer cell membrane, a characteristic feature of apoptotic cell death [24] and to quantify the percentage of apoptotic cell death, cultured MDA-MB-231 cells (1 × 10^6^) were treated with 50 % inhibitory concentration (30 μM) of andrographolide for 24 h, 36 h and 48 h. Cells were resuspended in 1X binding buffer, labelled with fluorescein isothiocyanate (FITC)-conjugated Annexin V (200 μg/ml) and PI (30 μg/ml), incubated for 15 min at room temperature in the dark and analyzed by flow cytometry using BD FACSVerse™ (Becton Dickinson, USA). Data were analyzed using FACSuite™ software. For each set, total of 10,000 events were recorded. Similar experiment was also performed using MCF-7 cells treated with andrographolide for 48 h.

### Measurement of cellular Reactive Oxygen Species (ROS) level

To estimate intracellular ROS level, MDA-MB-231 (2 × 10^4^ cells per well) were treated with andrographolide (15, 30, 45 and 60 μM) for 12–48 h and were harvested by centrifugation for the assay. The cell suspension (200 μl, containing 1 × 10^5^ cells per ml) was mixed to PBS (800 μl) and 10 μM 2ʹ, 7ʹ-dichlorofluorescein diacetate (DCF-DA) and kept at 37 °C for 30 min in the dark. 25 μM H_2_O_2_ was used for positive control. The intracellular ROS level was measured by determining the production of H_2_O_2_ using a spectrofluorometer (excitation, 488 nm; emission, 515 nm). Another set of experiment was similarly performed where MDA-MB-231 cells were pre-treated with 3 mM NAC for 1 h followed by without or with andrographolide (30 μM) treatment. Same experiment was also performed using MCF-7 cells.

### Measurement of mitochondrial membrane potential

The loss of mitochondrial membrane potential (∆*ψ*m) (MMP) is a primary event leading to phosphatidyl serine externalization, which corresponds with the activation of caspase. MMP was quantified by the incorporation of a fluorescent dye Rhodamine 123 [25]. Cells (1 × 10^4^ per well) were incubated with different concentrations (15, 30, 45 or 60 μM) of andrographolide in the absence or presence of NAC for 24 and 48 h. Positive control was obtained with a mitochondrial uncoupling agent, carbonyl cyanide m-chloro-phenylhydrazone (CCCP; 20 μM) for 30 min. The treated cells were washed twice with chilled PBS before incubation with Rhodamine 123 (5 μg/ml) and kept at 37 °C for 15 min. The fluorescence intensity was detected using an emission filter of 535 nm. Same experiment was also performed using MCF-7 cells.

### Cytosolic and mitochondrial fraction preparation

Cells were treated with andrographolide (30 μM) for 48 h and mitochondrial as well as cytosolic fractions were obtained with a mitochondria isolation kit (Pierce, Rockford, USA). The mitochondrial pellet was re-suspended in sample buffer for SDS-gel electrophoresis and analyzed by Western blot using cytochrome *c* antibody. COX IV and β-actin were used as loading controls for the mitochondrial fraction and cytosolic fraction respectively. Cytosolic fractions were also Western blotted for studying cytochrome *c* expression [26].

### Western blotting

Cells from control regime and andrographolide treatment (30 μM, 48 h) were used for Western blot [27]. After centrifugation, cell pellets were lysed in RIPA buffer containing protease and phosphatase inhibitors (Roche, Germany). Each cell lysate was used for protein assay with Bio-Rad protein assay kit (Bio-Rad, USA) [28]. A protein sample of 50 μg was electrophoresed in a 12 % SDS-PAGE. Proteins were electro-transferred from the gel onto a nitrocellulose membrane (Amersham Biosciences, UK). After incubating with blocking buffer containing 5 % nonfat milk for 1 h, the membranes were probed with the primary antibody specific for Bcl-xL (1:750 dilution), Bcl-2 (1:1000), Bax (1:1000), cytochrome *c* (1:800) and Apaf-1 (1:500) at 4 °C. Membrane was then washed thrice with TBST (Tris buffered saline having 0.1 % Tween-20, pH 7.5), exposed to horseradish peroxidase conjugated secondary antibody (1:2500) and was then kept at 25 °C for 1 h. The membrane was then finally washed thrice with TBST. The protein bands were visualized by incubating the membrane with diaminobenzidine/hydrogen peroxide (Sigma-Aldrich) for color reaction. β-Actin and COX IV were also detected as loading controls.

### Caspase activity assay

MDA-MB-231 cells (1 × 10^5^ cells per ml) were incubated with different concentrations of andrographolide (0, 15, 30, 45 and 60 μM) for 24 h and then centrifuged to detect caspase-3 and caspase-9 activation using caspase fluoremetric protease assay kit following manufacturer’s instructions. Briefly, cells were lysed in RIPA buffer supplemented with protease inhibitor cocktail and 10 μl lysate were diluted with 10 μl substrate buffer (1:1) containing 100 μM each of fluorogenic substrate, DEVD-AFC and LEHD-AFC for caspase-3 and caspase-9 respectively, and was then incubated for 90 min at 37 °C. Reactions were stopped using 0.2 mM sodium phosphate buffer (pH 7.5) and fluorescence was quantified by a spectrofluorometer (excitation, 405 nm; emission, 505 nm). Experiment was also performed using MCF-7 cells.

Similar set of experiment was designed with MDA-MB-231 cells treated with 30 μM andrographolide along with Ac-DEVD-CHO (caspase-3 inhibitor) or Ac-LEHD-CHO (caspase-9 inhibitor). DMSO treated cells were considered as control set and compared.

### In vivo pharmacokinetic studies

Female BALB/c mice, being divided into two groups (*n* = 6 per group), received andrographolide via oral route (PO) as well as intravenous (IV) bolus dosing at 50 mg/kg (equivalent to 142.73 μmol/kg and dose volume, 10 ml/kg) and 5 mg/kg body weight (equivalent to 14.27 μmol/kg and dose volume, 5 ml/kg) respectively. The formulation was prepared in 10 % DMSO, 10 % Cremophor and 80 % 1/15 (M) Na_2_HPO_4_ with dose volume of 10 ml/kg and 5 ml/kg for oral and IV respectively. ~50 μl of blood samples were collected at each time points (0.25, 0.5, 1, 2, 4, 8 and 24 h for PO and 0.08, 0.25, 0.5, 1, 2, 4, 8 and 24 h for IV) into heparinized capillary tubes by piercing the saphenous vein using a disposable needle (26 G). Blood samples were centrifuged at 3000 rpm for 10 min at 4 °C. The resulting plasma samples were stored at –80 °C until bioanalysis.

### LC-MS/MS analysis

Analysis of plasma samples were performed using a liquid chromatography tandem mass spectroscopy (API-4000, AB Sciex Instruments, Foster City, CA) attached with the ESI source. The LC system consisted of LC-20ADvp pump (Shimadzu, Kyoto, Japan) and CTC PAL (HTS) autosampler. Liquid chromatography was performed using a Luna C18 column (2 × 30 mm, 5 μ particle) of Phenomenex (Torrance, CA) and the mobile phase consisted of 2 mM ammonium acetate in water (A) and 80: 20 (v/v) MeCN: Solvent A (B). The gradient elution program was as follows: first 60 s only A for washing, then 60 s for gradient up to 100 % B and it was continued for next 60 s. Total run time was 3.5 min with a flow of 0.8 ml/min at room temperature. Samples were detected and quantified using Analyst 1.4.2. Internal standard used was diclofenac.

### Statistical analysis

The data were expressed as the means of three replications with standard deviation (± SD). GraphPad Prism5 software was used for analysis of variance (ANOVA) calculation. A *p*-value of <0.05 was considered as statistically significant.

## Results

### Effect of andrographolide on cell viability and proliferation

**Fig. 1:**
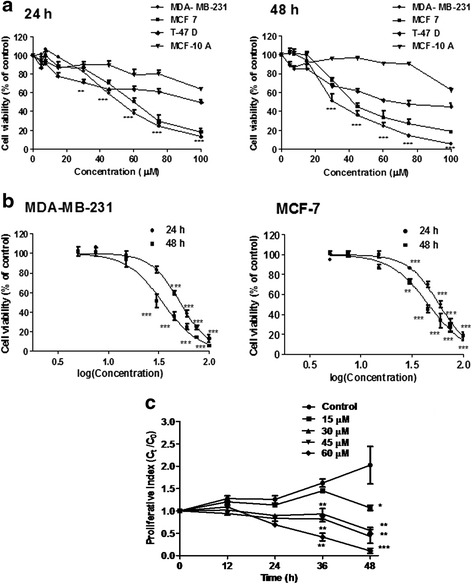
Assessment of cell viability in response to andrographolide. **a** Three breast cancer cell lines, MDA-MB-231, MCF-7, T-47D and a normal breast epithelial cell line MCF-10A were treated with andrographolide (5–100 μM) or vehicle control (0.1 % DMSO) for 24 h and 48 h. Time- and concentration- dependent inhibitory effects of andrographolide were evaluated by MTT assay. **b** The IC_50_ of andrographolide on MDA-MB-231 and MCF-7 were calculated by plotting the percentage of viable cells against log (concentration) of andrographolide. **c** Effect of andrographolide on proliferation kinetics of MDA-MB-231 cells. Cells (1 × 10^4^/well) in a 24-well culture plate were treated with varying concentrations of andrographolide for different time points. Cell proliferation was evaluated using trypan blue. Values are mean ± S.D. of three independent experiments (*P < 0.05, **P < 0.01 and ***P < 0.001)

For determining the effect of andrographolide on cell viability, three different breast cancer cell lines namely MDA-MB-231, T-47D and MCF-7 were incubated in the absence or presence of andrographolide (5–100 μM) for 24 or 48 h. MTT assay was performed to compare the cell viability with a normal breast epithelial cell line, MCF-10A. Andrographolide resulted in loss of cell viability in all three lymphoma cell lines (MDA-MB-231, T-47D and MCF-7) with the function of concentration and time of treatment (Fig. 1a). The degree of cytotoxicity on the selected cell lines varied, and it was found to be most potent on MDA-MB-231. The IC_50_ values at 24 and 48 h was found to be 51.98 μM and 30.28 μM respectively in MDA-MB-231(Fig. 1b). For MCF-7 cells, the IC_50_ values were estimated to be 61.11 μM (24 h) and 36.9 μM (48 h) respectively (Fig. 1b) while T-47D was found to be lesser sensitive at both the time points (Table 1). It was also observed that the same doses could not inhibit the proliferation of MCF-10A, normal human mammary epithelial cells to that extent. The calculated IC_50_ values were found to be 137.9 μM (24 h) and 106.1 μM (48 h) for MCF-10A cells which signifies that the IC_50_ at 48 h was nearly 3.5-fold higher than that for MDA-MB-231 cells. As andrographolide was found to be most cytotoxic towards MDA-MB-231 among the tested cell lines, therefore, we focused on the anticancer property of andrographolide in this TNBC cell line.

**Table 1:** Cytotoxic effects of andrographolide at IC_50_ (μM)^a^ on three breast cancer cell lines after different exposure time

Time (h)	MDA-MB-231	MCF-7	T-47D	MCF-10A
24	51.98	61.11	118.5	137.9
48	30.28	36.90	72.6	106.1

^a^IC_50_ = Concentrations corresponding to 50 % growth inhibition

Anti-proliferative potential of andrographolide was further confirmed by monitoring the proliferation kinetics of exponentially growing MDA-MB-231 cells. A dose- and time-dependent reduction in the cell proliferation rate was evident with a cytostatic effect up to 24 h post-treatment followed by growth inhibition (Fig. 1c) except in cells treated with 60 μM andrographolide. Cells treated with 15 μM of compound showed growth inhibition only after 48 h of treatment. ~50 % cell death was observed at a concentration of 30 μM at 48 h.

### Effect of andrographolide on the cell and nuclear structure of MDA-MB-231 cells

**Fig. 2:**
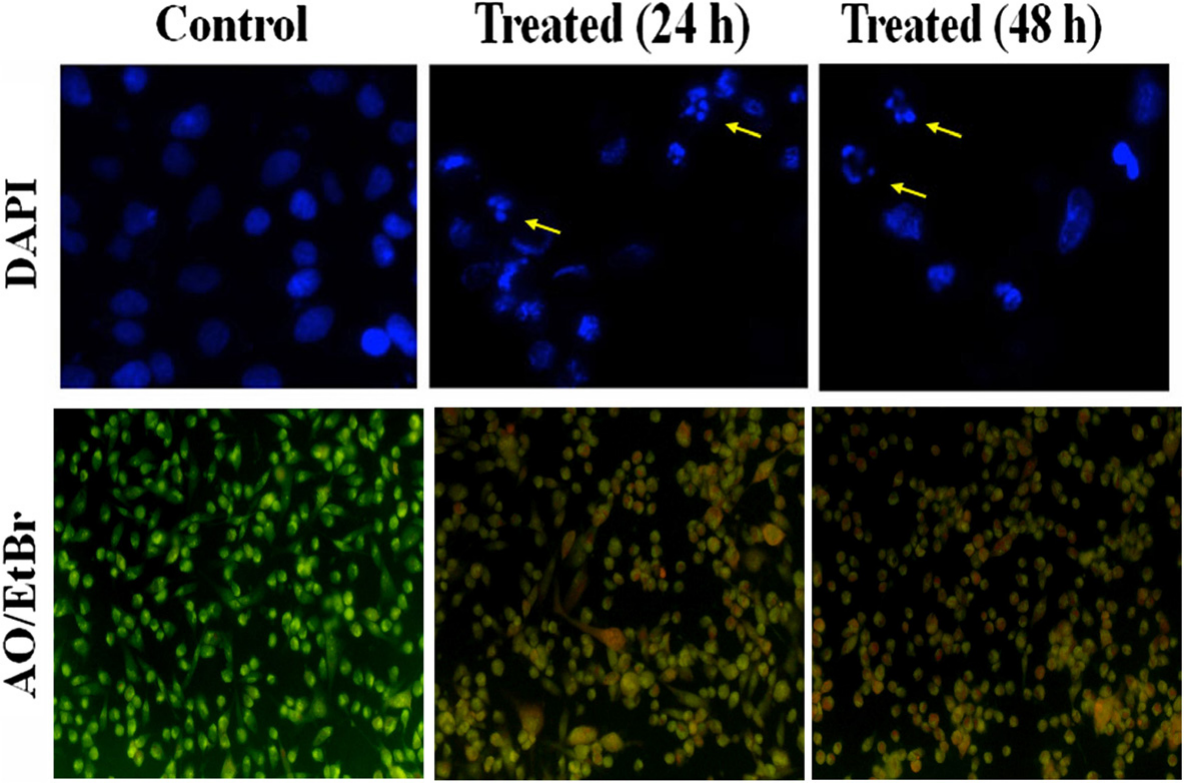
Assessment of cell morphology of MDA-MB-231 treated without or with andrographolide. *Upper panel* Effect of andrographolide in MDA-MB-231 cells with condensation and fragmentation of the nuclei identified by DAPI by Fluorescence microscopy. Cells were treated with IC_50_ concentration of andrographolide for 24 and 48 h and stained with DAPI. The fragmented apoptotic nuclei were shown by arrow. Data shown are from a representative of triplicate experiments. *Lower panel* Acridine orange/Ethidium bromide (AO/EtBr) staining of MDA-MB-231 cells after treatment of IC_50_ concentration of andrographolide for 24 and 48 h and compared with untreated control cells using fluorescence microscopy. Cells showing bright orange fluorescence indicate apoptosis in comparison to control cells showing green fluorescence

Involvement of apoptosis in andrographolide induced cytotoxicity in MDA-MB-231 at IC_50_ dose was examined using fluorescent DNA binding dye DAPI. Staining with DAPI revealed characteristic apoptotic changes like chromatin condensation, nuclear pyknosis, elevated number of nuclear body fragments and irregular edges around the nucleus in treated cells after 24 and 48 h treatment; while round, clear edged, uniformly stained cell nuclei were noted in the untreated control (Fig. 2, upper panel).

When andrographolide treated MDA-MB-231 cells were stained with AO/EtBr dye mixture, a major proportion of cells showed condensed or fragmented chromatin with bright orange fluorescence indicating apoptosis in comparison with the control cells which showed green fluorescence (Fig. 2, lower panel). The results indicated that death induced by andrographolide of MDA-MB-231 cells was due to apoptosis.

### Effect of andrographolide on cell cycle of MDA-MB-231 cells

**Fig. 3:**
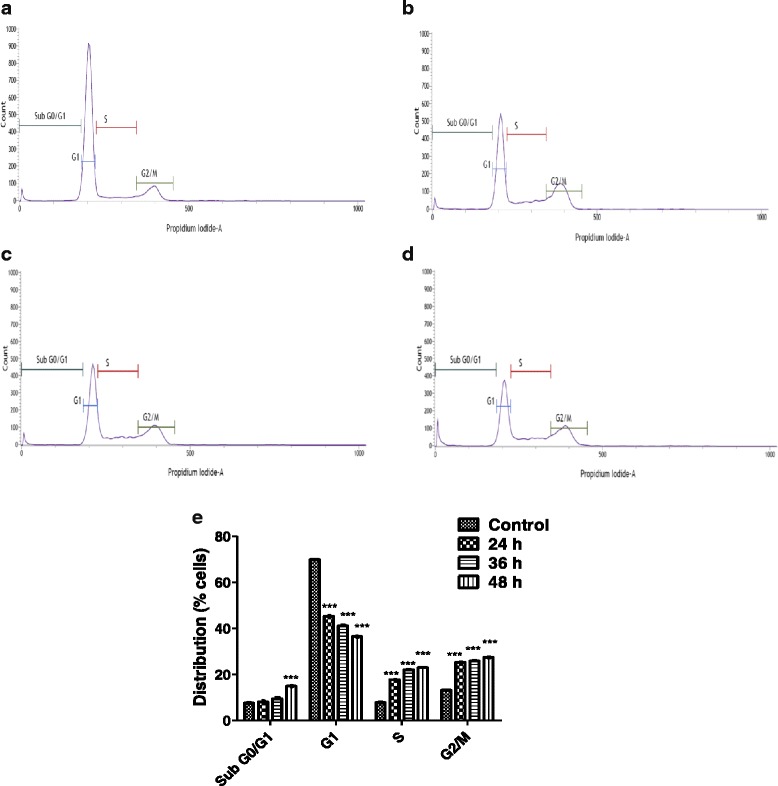
Cell cycle histograms of the DNA content of MDA-MB-231 in response to andrographolide. Cells were incubated for 24 (**b**), 36 (**c**) and 48 (**d**) h time points in the absence (control, 48 h) (**a**) or presence of andrographolide (30 μM), prepared for FACS analysis, as described in Materials and Methods, and analyzed in FACSVerse™ (Becton Dickinson, USA) flow cytometer. The number of cells in each phase of the cell cycle was obtained by using FACSuite™ software. **e** The percentage of cells in each phase was shown as the mean ± S.D. from three independent experiments (*P < 0.05, **P < 0.01 and ***P < 0.001)

Inhibition of cell cycle progression with 30 μM andrographolide was evaluated at different time points in MDA-MB-231 cells. Andrographolide treatment (30 μM) showed an increasing trend in the cell number at S phase of MDA-MB-231 cells with time and decreasing trend in the cell number at G_1_/G_0_ phase (Fig. 3). The increase in S population after 24 and 36 h of treatment were found to be 9.91 ± 0.3 % and 14.21 ± 0.2 %, respectively over the untreated control set. There was no significant change in cell cycle arrest profile beyond 36 h. In each treatment regime, a notable decrease in the cell number at the G_1_/G_0_ phase was observed; quantitatively it was 24.75 ± 0.5 %, 28.86 ± 0.7 % and 33.53 ± 1.1 % for 24, 36 and 48 h of treatment respectively over the control set. It was also evident that the S phase arrest was accompanied with an increase of 12–14 % in G_2_/M phase cells irrespective of increase of time points.

### Andrographolide causes externalization of phosphatidyl serine and apoptosis

**Fig. 4:**
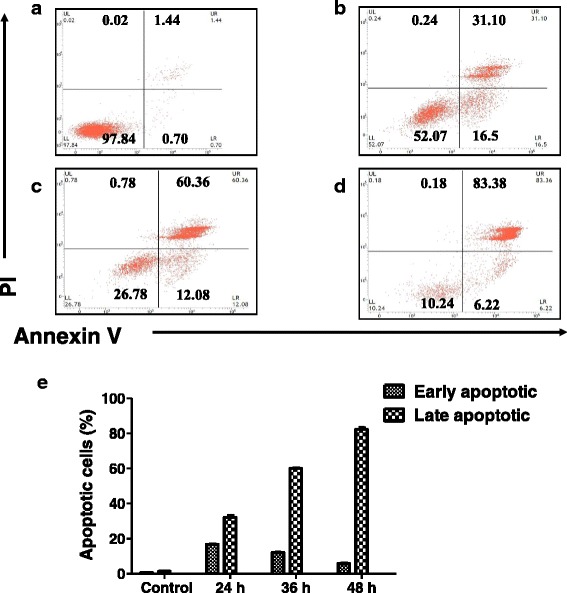
Flow cytometry analysis of andrographolide induced apoptosis in MDA-MB-231 cells. Cells were double stained with annexin V-FITC and PI after treatment with andrographolide (30 μM) for 24 (**b**), 36 (**c**) and 48 (**d**) h and analyzed in a FACSVerse™ (Becton Dickinson, USA) flow cytometer. Untreated cells for 48 h served as Control (**a**). The proportions of early apoptotic (Annexin V^+^ PI^-^) & late apoptotic cells (Annexin V^+^ PI^+^) were significantly increased following andrographolide exposure compared to Control. **e** Bar diagram showing increased proportion of early and late apoptotic population after andrographolide treatment. Data are representative of three independent experiments

The percentage of apoptotic cells in andrographolide treated MDA-MB-231 cells were monitored using FITC-Annexin V and propium iodide (PI) double staining by FACS. As shown in Fig. 4, andrographolide exposure for 24, 36 and 48 h resulted in 16.76 ± 0.51 %, 12.23 ± 0.29 % and 5.89 ± 0.67 % early apoptotic cells (annexin V^+^ and PI^-^) respectively compared to control (<1 %), and the amount of late apoptotic cells (annexin V^+^ and PI^+^) were 32.2 ± 2.2 %, 60.06 ± 0.6 % and 82.41 ± 1.9 % for the same treatment respectively. Untreated cells contained only 1.4 ± 0.08 % late apoptotic cells. Less than 1 % of cell population underwent necrotic phase in all the treated conditions.

Effect of andrographolide on apoptosis in MCF-7 cells was also determined. Treatment for 48 h with andrographolide increased the percentage of apoptotic cells from 2.01 % in the control to 41.17 % (Additional file 1), which indicated that andrographolide could much efficiently induce apoptosis in MDA-MB-231, triple negative breast cancer cells compared to MCF-7.

### Andrographolide induces ROS accumulation and reduces MMP

**Fig. 5:**
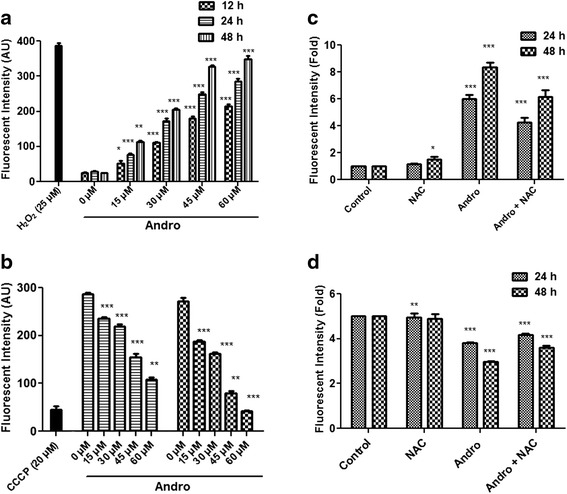
Effect of different concentrations of andrographolide on production of ROS and MMP in MDA-MB-231 cells. **a** Cells were treated with different concentrations (0–60 μM) of andrographolide and incubated for 12, 24 and 48 h. 25 μM H_2_O_2_ was used as a positive control. Cells were stained with 2′,7′-dichlorofluorescein diacetate and the production of intracellular H_2_O_2_ was measured using a spectrofluorometer as described in materials and methods. Values are mean ± S.D. and represent one of the three independent experiments (*P < 0.05, **P < 0.01 and ***P < 0.001). **b** Effect of andrographolide on mitochondrial transmembrane potential (∆*ψ*m) in MDA-MB-231 cells after treatment with different concentrations (0–60 μM) of compound for 24 and 48 h. An uncoupling agent CCCP (20 μM) was served as positive control. MMP was measured by spectrofluorometer using a fluorescent probe Rhodamine 123. Values are mean ± S.D. and represent one of the three independent experiments (*P < 0.05, **P < 0.01 and ***P < 0.001). **c**-**d** MDA-MB-231 cells were pretreated with or without 3 mM NAC for 1 h followed by 30 μM andrographolide for 24 and 48 h and quantified for ROS production (**c**) and loss of ∆*ψ*m (**d**). Fold changes were determined relative to control. Values are mean ± S.D. and represent one of the three independent experiments (*P < 0.05, **P < 0.01 and ***P < 0.001)

Intracellular ROS generation was found to be related to different stresses and could contribute on cell cycle arrest or cellular apoptosis. To examine whether andrographolide affects the oxidative function of the cell, ROS generation was quantified in MDA-MB-231 at different time points. Cells were loaded with the permeable and redox-sensitive dye, DCF-DA. ROS production due to andrographolide treatment (15, 30, 45 and 60 μM) was measured at different time periods. Untreated set was considered as control. ROS generation was found to be at the basal level in untreated cells. On the contrary, andrographolide treatment resulted increasing ROS generation with longer exposure and elevated dose as reflected by increasing DCF fluorescence (Fig. 5a). Similar dose- and time-dependent effect on ROS generation was also found in MCF-7 cells (Additional file 2: Figure S2A).

The effect of andrographolide on MMP in MDA-MB-231 cells was also investigated to determine whether andrographolide induced-ROS production coincides with loss of MMP (∆*ψ*m), which is considered as an early intracellular event during onset of apoptosis. MMP was determined by incorporating a cationic fluorescent dye Rhodamine 123. Andrographolide caused a significant (*P* < 0.01) drop of MMP level with increasing concentrations of compound in MDA-MB-231 cells at 24 and 48 h (Fig. 5b). Moreover, pretreatment of cells with N-acetyl cysteine (NAC), an antioxidant, suppressed andrographolide-induced ROS generation (Fig. 5c) and loss of ∆*ψ*m (Fig. 5d) at 24 as well as 48 h. Taken together, these data suggest that induction of apoptosis by andrographolide is likely to be mediated by increased ROS production and loss of ∆*ψ*m leading to an oxidative stress-dependent manner of apoptosis in MDA-MB-231 cells. We also tested the effect of andrographolide on MCF-7 cells and observed similar dose- and time-dependent effect on MMP loss (Additional file 2: Figure S2B).

### Effect of andrographolide on cellular protein expression and release of mitochondrial cytochrome c

**Fig. 6:**
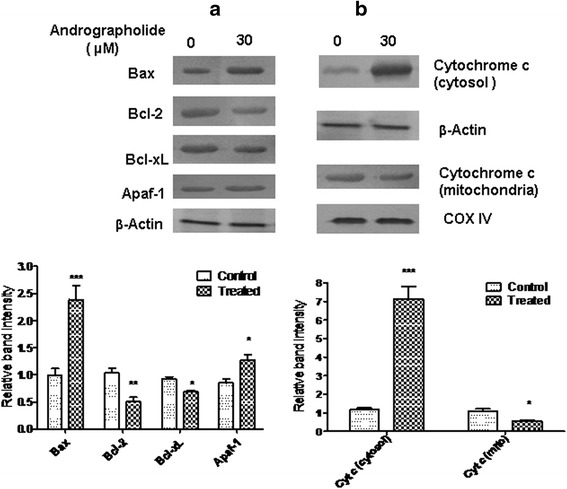
Western blot analysis of some important apoptosis associated proteins in MDA-MB-231 cells. Cells were treated with vehicle control (0.1 % DMSO) or with andrographolide (30 μM) for 48 h. Cells were then lysed and cell lysate were used for Western blot analysis as described in materials and methods. Data shown are representatives of three independent experiments. **a** Western blot analysis of different pro- and anti-apoptotic proteins. Expressions of Bax, Bcl-2, Bcl-xL and Apaf-1 in total cell lysate were detected after treatment. Band intensities were quantified by ImageJ and normalized to β-Actin which was used as loading control. Data were expressed as a band intensity relative to control and shown below [means ± S.D., *n* = 3; *P < 0.05, **P < 0.01, ***P < 0.001 compared with control]. **b** Effect of andrographolide on expression of cytochrome *c* in cytosolic and mitochondrial fraction in Western blot analysis. β-Actin and COX IV were detected as loading control respectively. Band intensities were quantified by ImageJ and normalized to respective loading controls. Data were expressed as a band intensity change compared to control and shown below [means ± S.D., *n* = 3; *P < 0.05, **P < 0.01, ***P < 0.001 compared with control]

The expression of various pro- and anti-apoptotic proteins in MDA-MB-231 cells was investigated in andrographolide-treated and untreated cells by Western blot. The expression level of Bcl-xL, an anti-apoptotic protein was significantly down-regulated to 1.47 ± 0.06 fold compared to control after treatment with 50 % inhibitory dose (30 μM) of andrographolide when exposed for 48 h (Fig. 6a). An attempt was made to find the state of proportion between cellular Bax and Bcl-2. The treatment significantly up-regulated pro-apoptotic protein Bax expression (by 2.39 ± 0.45 fold) and down regulated anti-apoptotic protein Bcl-2 expression (by 1.96 ± 0.14 fold) in MDA-MB-231 cells and as a result Bax/Bcl-2 ratio was found to be increased (Fig. 6a). The expression of another pro-apoptotic protein, Apaf-1 was also found to increase by 1.49 ± 0.17 fold (Fig. 6a). Interestingly, we also observed a highly significant increase in the expression (by 7.13 ± 1.17 fold) of cytosolic cytochrome *c* whereas decrease in the expression of cytochrome *c* present in the mitochondrial fraction (1.92 ± 0.05 fold) (Fig. 6b). Taken together, these data suggest that andrographolide induced changes in Bax/Bcl-2 ratio might trigger the release of cytochrome *c* from mitochondria and enhance the deposition in cytoplasm which could bind to Apaf-1 and subsequently lead to apoptosis.

### Effect of andrographolide on caspase activities

**Fig. 7:**
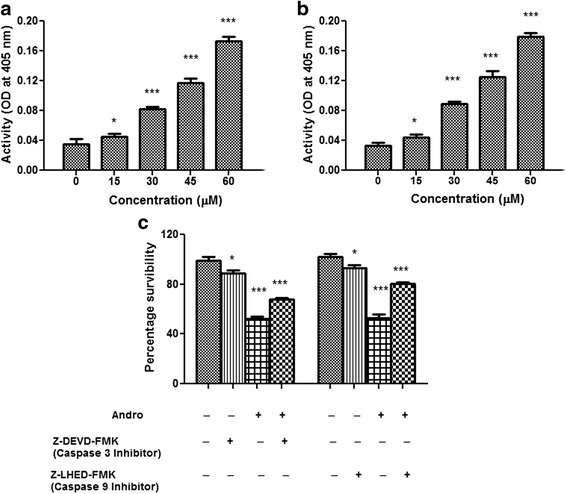
Caspase activity assay in MDA-MB-231 cells. Treated (0–60 μM) and untreated cells for 24 h were tested for fluoremetric assay to determine the levels of activated caspase-9 and caspase-3. **a** caspase-9 and **b** caspase-3 activities were enhanced by andrographolide dose dependently. **c** Cells were treated with andrographolide, or andrographolide + caspase inhibitors (50 μM) at the indicated dose and time period. All of the caspase inhibitors significantly inhibited andrographolide-induced apoptosis. All data represent mean ± S.D. from 3 independent experiments (*P < 0.05, **P < 0.01 and ***P < 0.001)

Molecular events leading to apoptosis in MDA-MB-231 cells were further tested by measuring activation of caspase-9 and caspase-3 in cells treated without (control) or with increasing concentrations of andrographolide. The results illustrated a gradual dose-dependent activation of caspase-9 (Fig. 7a) and caspase-3 (Fig. 7b). Andrographolide-induced cell death became lowered when the cells were pretreated with the caspase inhibitors Ac-LEHD-CHO and Ac-DEVD-CHO (Fig. 7c). This finding demonstrates that andrographolide might induce activation of intrinsic caspase pathway in MDA-MB-231 cells. Besides, caspase-9 (initiator caspase) and caspase-7 (effector caspase) activation in a dose-dependent manner were observed in caspase-3 deficient MCF-7 cells after treatment with andrographolide (Additional file 3).

### In vivo pharmacokinetics: plasma levels of andrographolide

The concentrations of test samples were determined from the standard curve prepared by spiking 2 μl of working stock in 98 μl of blank plasma along with the quality control samples (low, mid and high). The linear equation of the standard curve was obtained by regressional analysis of the peak area ratio of analyte to internal standard versus nominal concentration with a weighting factor of 1/*X*2. The calibration curve was linear in the concentration range 2.44 (LLOQ) to 1250 ng/ml.

**Fig. 8:**
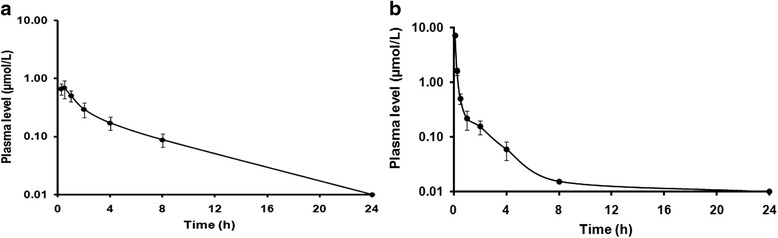
Plasma concentration of andrographolide vs. time following oral administration of a single dose of 50 mg/kg (**a**) as well as IV bolus dose of 5 mg/kg (**b**) in female BALB/c mice (mean ± S.D., *n* = 6)

In our study, the pharmacokinetic parameters of andrographolide were calculated based on non-compartmental model using Win-Nonlin 6.3. The % bioavailability (BA) of andrographolide was determined after PO (50 mg/kg) and IV (5 mg/kg) administration (Fig. 8a, b). AUC is one of the fundamental PK parameters to correlate plasma exposure with oral bioavailability of a drug. The results of PK study revealed poor oral bioavailability (9.27 ± 1.69 %) of andrographolide with a C_max_ of 0.73 ± 0.17 μmol/L and T_max_ of 0.42 ± 0.14 h following oral administration (Table 2). Andrographolide showed moderate terminal half lives (T_1/2_) of 1.86 ± 0.21 and 3.30 ± 0.35 h following IV and oral administration respectively. This compound was also observed to be rapidly metabolized and quickly eliminated from the central compartment with a high clearance (CL) of 5.51 ± 1.09 L/h/kg after IV administration while the total exposure (AUC_0_–α) was found to be 2.33 ± 0.42 μmol.h/L and 2.51 ± 0.56 μmol.h/L following oral and IV administration respectively.

**Table 2:** Pharmacokinetic parameters calculated following oral administration (PO: single dose of 50 mg/kg body weight) and intravenous administration (IV: single dose of 5 mg/kg body weight) of andrographolide in female BALB/c mice

PK parameters	PO at 50 mg/kg	IV at 5 mg/kg
AUC_(0-8)_ (μmol.h/L)	1.92 ± 0.36	1.96 ± 0.38
AUC_(0-∞)_ (μmol.h/L)	2.33 ± 0.42	2.52 ± 0.56
C_max_ (μmol/L)	0.73 ± 0.17	ND
T_max_ (h)	0.42 ± 0.14	ND
C_0_ (μmol/L)	ND	14.57 ± 4.04
CL (L/h/kg)	62.68 ± 12.10	5.52 ± 1.09
Terminal T_½_ (h)	3.30 ± 0.35	1.87 ± 0.21
Bioavailability (%)	9.27 ± 1.69	ND

ND = Not determined; AUC_(0-8)_ is the total area under the curve from time 0 to 8 h; AUC_(0-∞)_ is the total area under the curve from time 0 extrapolated to infinite time combined with an extrapolated value; C_0_ is the drug concentration at time 0; CL is the drug clearance; Terminal T_1/2_ is the time for concentration to decrease by one-half in the elimination phase; C_max_ is the maximum concentration observed; T_max_ is the time at maximum concentration observed; Bioavailability is the fraction of administered drug that reached the systemic circulation

## Discussion

While radio- and/or chemotherapeutic treatments are effective tools in treating certain cancers, a small population of cancer stem cells (CSC) can evade therapy which could be the reason for tumor recurrence and high rate of mortality. These CSC have the ability to bring the existence of new tumors and these are frequently found as multi-drug resistance (MDR) [29]. Chemotherapeutic drugs theoretically target the metastatic sites but current treatments do not exert significant therapeutic benefits in all cases [30]. Thus care should be taken to develop an alternative therapeutic strategy. Plant derived anticancer drugs are being considered more effective and safer for the patients, and do not have significant side effects compared to synthetic drugs [31].

Considering public health due to increasing death rate resulting from cancer, hunt for natural products for cancer therapy is now being considered as a global challenge; many of which are in different phases of therapeutic trials [32]. According to a report of World Health Organization, currently ~80 % of world population relies on herbal medicine as its primary health care need [33, 34]. In India, use of herbs as traditional medicine is as old as 4000 BC [35]. Natural products were being used from ancient times in the treatment of cancer and interestingly more than 60 % of currently used cancer therapeutants are reported to be from the natural sources [36].

Bioactive principles from *A. paniculata* have attracted a lot of attention due to their anti-cancer and immuno-stimulatory properties and several reports are available on andrographolide-induced cellular apoptosis in different cell lines [11, 37]. This study reports on in vitro time-dependent and dose-dependent inhibitory effect of andrographolide on the proliferation and apoptosis of the human MDA-MB-231, a metastatic breast carcinoma cell line. The molecular mechanism of andrographolide induced apoptosis has also been partially elucidated in this clinically important cell line. Andrographolide was primarily examined for its cytotoxicity effects by MTT assay with MDA-MB-231 and with two other breast carcinoma cell lines. The results indicate that while andrographolide shows the most potent inhibitory effect on the survival of MDA-MB-231 cells with an IC_50_ of 30 μM at 48 h, it has a minimal effect on MCF-10A, a normal human breast epithelial cell line. Andrographolide exhibits anti-proliferative activity on these cells in a dose- and time-dependent manner. Further, andrographolide induces cellular apoptosis via changes in cellular morphology and occurrence of chromatin condensation, a hallmark of apoptosis, as reflected in DAPI and AO/EtBr staining. These results exhibit that andrographolide can restrain proliferation and stimulate nuclear DNA fragmentation resulting in augmentation of apoptosis in MDA-MB-231 cells. Additionally, flow cytometry with Annexin V/PI staining was conducted to detect apoptotic mode of cell death and to quantify apoptotic cells. It is evident from the result that initiation of andrographolide-induced phosphatidyl serine externalization took place after 24 h of treatment and subsequently increased upon exposure time. The number of apoptotic cells increased from 32 % after 24 h of exposure to 81 % after 48 h of exposure. This result further authenticated that andrographolide could inhibit the growth of breast cancer cells and subsequently induce apoptosis.

DNA damage is a molecular event which is closely associated with cell cycle arrest and apoptosis. In this study, andrographolide treatment resulted in an increase of cell population at S/G_2_M phase which might be due to the blockage in the cell cycle process from S phase to G_2_/M phase, thus arresting mitosis and making the cell cycle halted at S phase resulting in cellular apoptosis [38, 39]. To date, several studies showed that andrographolide effectively induces cell cycle arrest at G_0_/G_1_ stage in most of the cancer cells [40, 41], whereas our findings with regard to cell cycle arrest at the G_2_/M phase in MDA-MB-231 is novel. It may be due to different cyclin-dependent kinase (CDKs) activities being regulated with the association of their cyclin partners, kinases, phosphatases and specific inhibitors [42]. Further research is needed to examine the detail mechanism of cell cycle arrest in andrographolide-treated MDA-MB-231 breast cancer cells.

Mitochondrial damage has been reported as an early event of apoptosis [43] and is persistent with intracellular reactive oxygen species (ROS) generation and changes in mitochondrial membrane potential (MMP) taking a crucial part in induction of apoptosis [44]. Several studies have suggested that increased amount of ROS initiates the depolarization of MMP resulting in the release of cytochrome *c* which activates the apoptosome and therefore the caspase cascade [45]. This study reveals that andrographolide treatment significantly promotes ROS levels and loss of MMP in a time- and dose-dependent manner. Furthermore, pretreatment of MDA-MB-231 cells with an antioxidant NAC attenuates andrographolide-induced intracellular ROS production and loss of MMP, thus suggesting that oxidative stress can induce apoptosis in the breast cancer cells. Decreasing MMP may lead to the change in expression of the anti-apoptotic and pro-apoptotic effectors which involve a large number of proteins [46]. Therefore, to gain insight into the mechanism controlling apoptosis, the effect of andrographolide on anti-apoptotic Bcl-2 family members, such as Bcl-2 and Bcl-xL, as well as the pro-apoptotic member, Bax was also investigated. Results obtained from Western blotting analysis for Bax, Bcl-xL and Bcl-2 proteins in this study corroborates with the fact that the amount of pro-apoptotic protein Bax have been up regulated along with down regulation of anti-apoptotic protein Bcl-2 and Bcl-xL. Increase in Bax/Bcl-2 ratio leads to the activation of intrinsic mitochondria-mediated apoptotic pathway which is associated with the release of cytochrome *c* from mitochondrial membrane to cytosol, formation of Apaf-1/cytochrome *c* complex that facilitates the formation of apoptosome, activation of caspase 9 and consequently activation of caspase 3 [47]. The obtained data clearly shows that treatment with andrographolide markedly increased the expression of Apaf-1 in addition to **cytosolic cytochrome*c*** along with a significant reduction of the same in mitochondrial fraction.

**Fig. 9:**
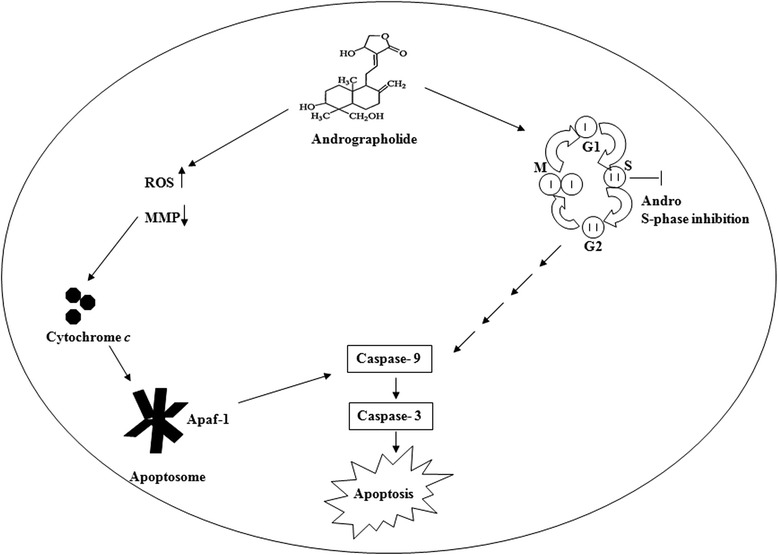
A proposed mechanism for apoptotic effects of andrographolide on the triple negative human breast cancer MDA-MB-231 cells

The activation of cysteine aspartate- specific proteases (caspases) is generally considered to be one of the key events in apoptosis pathway. A classical apoptotic mode of cell death occurs only if the execution of death depends on caspase activity [48]. Caspases can be broadly divided into the group of upstream initiator caspases including caspases -8 and -9, and into the group of downstream executioner caspases including caspases-3 [49]. Caspase-dependent pathway can further be subdivided into extrinsic or intrinsic pathway, depending on the role of caspase-8 or caspase-9, respectively. It seems that apoptotic signaling elicited by andrographolide might be related to the mitochondrial pathway, thus caspase-9 and caspase-3 activation was studied during the induction of apoptosis. We observed activation of caspase-9 and caspase-3 after treatment of andrographolide. Inhibitors specific to these caspases could restore the cells from cell death. From these observations, it may be suggested that once activated, this initiator caspase (caspase-9) can cleave and stimulate caspase-3 and other effector caspases, thereby turning on a cascade of events expediting DNA fragmentation and cell death [50]. Thus, the findings, as schematically presented in Fig. 9, indicate that accumulation of ROS, up-regulation of Bax, down-regulation of Bcl-2 and Bcl-xL, Apaf-1/cytochrome *c* apoptosome development and activation of caspase-9 and -3 may be the molecular mechanism by which andrographolide induces apoptosis in the highly metastatic MDA-MB-231 cells which are ER-negative and contains mutated p53. Other researchers also have reported that andrographolide inhibits cell proliferation and induces apoptosis in breast cancer cells (like MCF-7) which are ER-positive and possess wild type p53 [16, 37]. Earlier, Satyanarayana et al. [17] reported that andrographolide could block the cell cycle at G_0_-G_1_ phase on MCF-7 cells. Our findings for andrographolide in MCF-7 also indicate that this agent is capable of enhancing ROS accumulation, loss of MMP, externalization of phosphatidyl serine and activation of caspase-9 and -7. Current observations suggest that the mode of action is primarily similar in MCF-7 cells to that of MDA-MB-231 except the cell cycle phase arrest. Therefore, it could be concluded that the effects of andrographolide on inhibition of cell proliferation and induction of apoptosis are irrespective of ER and p53 status.

In vivo pharmacokinetics study with andrographolide showed that it is rapidly absorbed in plasma although it has poor bioavailability in mice (<10 %). The observed reduced AUC from zero to 8 h was probably due to relatively rapid elimination of andrographolide from the body. Plasma exposure at 24 h was below lower limit of quantitation in both PO and IV arms. The total exposure AUC_0-8_ and AUC_0-_α were 1.92 and 2.33 μmol.h/L respectively after oral administration. Terminal half life of andrographolide in PO arm was two times higher than IV arm. All these parameters performed in mice indeed correlate with the values obtained from earlier studies performed in rats [51]. Therapeutic potency of any drug depends on its bioavailability and poor solubility of andrographolide in water affects its bioavailability. However, it was evident from previous reports that andrographolide is extremely nontoxic even at high doses [7, 52]. The LD_50_ of andrographolide in male mice was noted to be 11.46 g/kg via intraperitoneal route [53]. Andrographolide showed no cytotoxic effect on platelets after administering it at concentrations between 35 and 150 mM [54]. Moreover, an inclusion technique has recently been developed to transform its physical and chemical properties so as to increase its bioavailability and prevent its degradation in neutral and alkaline environment of gastrointestinal tract [55]. It has been shown that, when taken as Kan Jang tablets, a therapeutic formulation of *A. paniculata* extract, andrographolide is quickly absorbed in blood [56]. Besides, drug discovery of andrographolide analogues can lead to an effective way out in this context.

On that basis along with our results, andrographolide seems to have a potential role as an ideal chemopreventive agent for breast cancer although it deserves further in-depth studies in order to explore its precise mechanism of action as an anticancer agent in vivo.

## Conclusions

These findings confirm that andrographolide induces apoptosis effectively in the mutant p53, triple negative MDA-MB-231 human mammary epithelial carcinoma cells in vitro. It has anti-proliferative activity through the mitochondria dependent caspase mediated pathway. Cell cycle arrest at G_2_/M phase by andrographolide is a major finding of this work. Moreover, our work establishes that ROS, MMP and caspase-3 & -9 are the known key players involved in andrographolide induced apoptosis. It seems that andrographolide might be a good contender as cancer therapeutant from a natural source for human breast cancer. It also warrants further in-depth investigation at pre-clinical and clinical levels for establishing it as a potential agent for cancer therapy and a toxicological study in higher animals and humans is also essential to design a scientifically defensible fact profile for the purpose of risk assessment prior to formulation.

## Additional files

Additional file 1: Andrographolide-induced externalization of phosphatidyl serine and apoptosis in MCF-7 cells. Figure S1. Effect of andrographolide treatment on apoptosis in MCF-7 cells. Cells were treated with IC_50_ concentration of andrographolide for 48 h, double stained with annexin V-FITC/PI and analyzed in a FACSVerse™ (Becton Dickinson, USA) flow cytometer. The percentage Annexin V-positive population refers apoptosis induction (region 2 and 4). Data are representative of three independent experiments. (PDF 88 kb) Additional file 2: Andrographolide-induced ROS accumulation and MMP loss in MCF-7 cells. Figure S2. (A) Effect of andrographolide treatment on ROS generation in MCF-7 cells. Cells were pretreated with or without 5 mM NAC for 1 h followed by different concentrations (0, 20, 40, 60 and 80 μM) of andrographolide and incubated for the indicated times (24 h and 48 h). Intracellular ROS production was monitored by spectrofluorometer, using 2′, 7′-dichlorofluorescein diacetate (DCF-DA). (B) Loss of MMP (∆*ψ*m) in MCF-7 cells upon treatment with andrographolide (0–80 μM) in presence and absence of NAC. MMP was measured by spectrofluorometer using a fluorescent probe Rhodamine 123. Results shown are representative of three independent experiments. **P* < 0.05, ***P* < 0.01 and ****P* < 0.001, when compared with control. (PDF 116 kb) Additional file 3: Effect of andrographolide on caspase-9 and caspase-7 activities in MCF-7 cells. Figure S3. Activation of caspase-9 (A) and caspase-7 (B) after treatment with different concentrations (0, 20, 40, 60 and 80 μM) of andrographolide in MCF-7 cells for 24 h. Results shown are representative of three independent experiments. **P* < 0.05, ***P* < 0.01 and ****P* < 0.001, when compared with control. (PDF 43 kb)

## Abbreviations

DAPI: 4, 6-diamidino-2-phenylindole
DMEM: Dulbecco’s Modified Eagle’s Medium
FBS: fetal bovine serum
IV: intravenous
PI: propidium iodide
MMP: mitochondrial membrane potential
MTT: 3-(4,5-dimethylthiazol-2-yl)-2,5-diphenyltetrazoliumbromide
NAC: N-acetyl cysteine
ROS: reactive oxygen species
SDS-PAGE: sodium dodecyl sulfate-polyacrylamide gel electrophoresis
