# Supplementary material for: Cytotoxicity and cell cycle arrest induced by andrographolide lead to programmed cell death of MDA-MB-231 breast cancer cell line
Source: J Biomed Sci. 2016 Apr 16;23:40. doi: 10.1186/s12929-016-0257-0 (PMC4833932; doi:10.1186/s12929-016-0257-0)
Supplement: Additional file 1: — Andrographolide-induced externalization of phosphatidyl serine and apoptosis in MCF-7 cells. Figure S1. Effect of andrographolide treatment on apoptosis in MCF-7 cells. Cells were treated with IC50 concentration of andrographolide for 48 h, double stained with annexin V-FITC/PI and analyzed in a FACSVerse™ (Becton Dickinson, USA) flow cytometer. The percentage Annexin V-positive population refers apoptosis induction (region 2 and 4). Data are representative of three independent experiments. (PDF 88 kb) [file 12929_2016_257_MOESM1_ESM.pdf]

Additional File 1

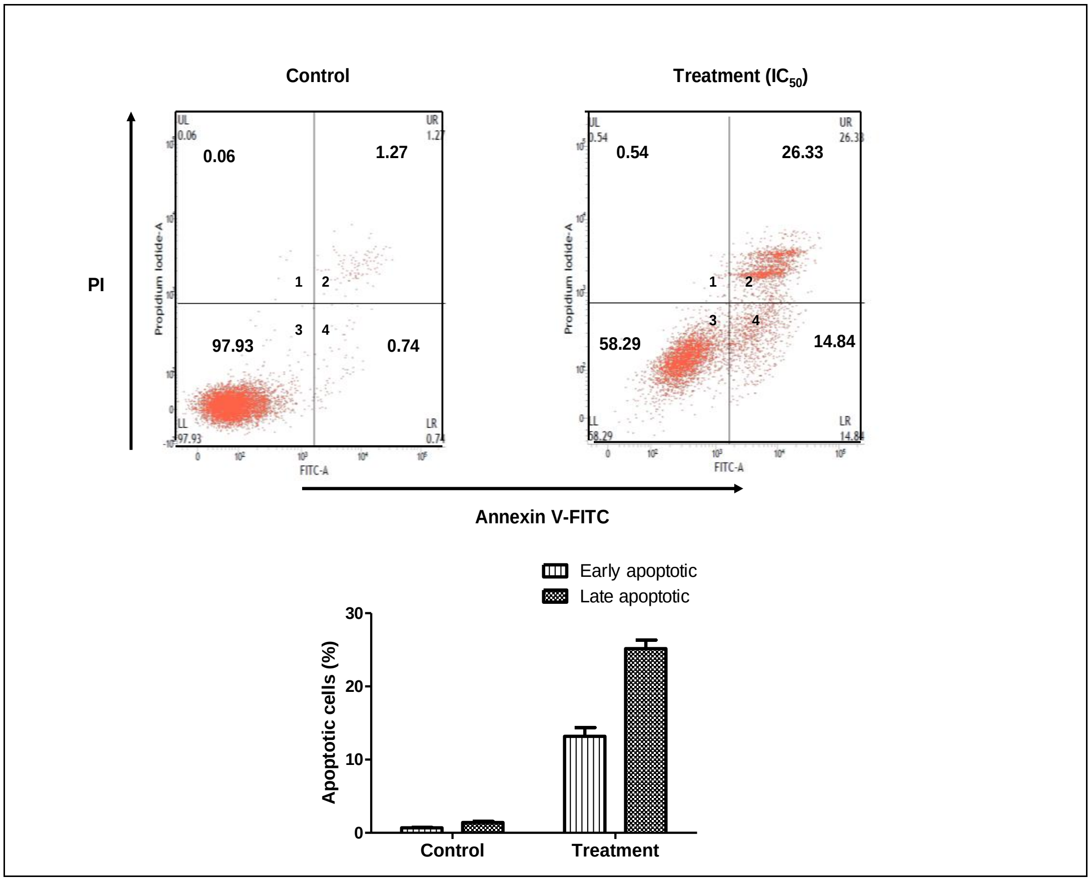

Figure S1. Effect of andrographolide treatment on apoptosis in MCF-7 cells. Cells were treated with IC_{50} concentration of andrographolide for 48 h, double stained with annexin V-FITC/ PI and analyzed in a FACSVerse™ (Becton Dickinson, USA) flow cytometer. The percentage Annexin V-positive population refers apoptosis induction (region 2 and 4). Data are representative of three independent experiments**.**
