# Supplementary material for: Cytotoxicity and cell cycle arrest induced by andrographolide lead to programmed cell death of MDA-MB-231 breast cancer cell line
Source: J Biomed Sci. 2016 Apr 16;23:40. doi: 10.1186/s12929-016-0257-0 (PMC4833932; doi:10.1186/s12929-016-0257-0)
Supplement: Additional file 2: — Andrographolide-induced ROS accumulation and MMP loss in MCF-7 cells. Figure S2. (A) Effect of andrographolide treatment on ROS generation in MCF-7 cells. Cells were pretreated with or without 5 mM NAC for 1 h followed by different concentrations (0, 20, 40, 60 and 80 μM) of andrographolide and incubated for the indicated times (24 h and 48 h). Intracellular ROS production was monitored by spectrofluorometer, using 2′, 7′-dichlorofluorescein diacetate (DCF-DA). (B) Loss of MMP (∆ψm) in MCF-7 cells upon treatment with andrographolide (0–80 μM) in presence and absence of NAC. MMP was measured by spectrofluorometer using a fluorescent probe Rhodamine 123. Results shown are representative of three independent experiments. *P < 0.05, **P < 0.01 and ***P < 0.001, when compared with control. (PDF 116 kb) [file 12929_2016_257_MOESM2_ESM.pdf]

Additional File 2

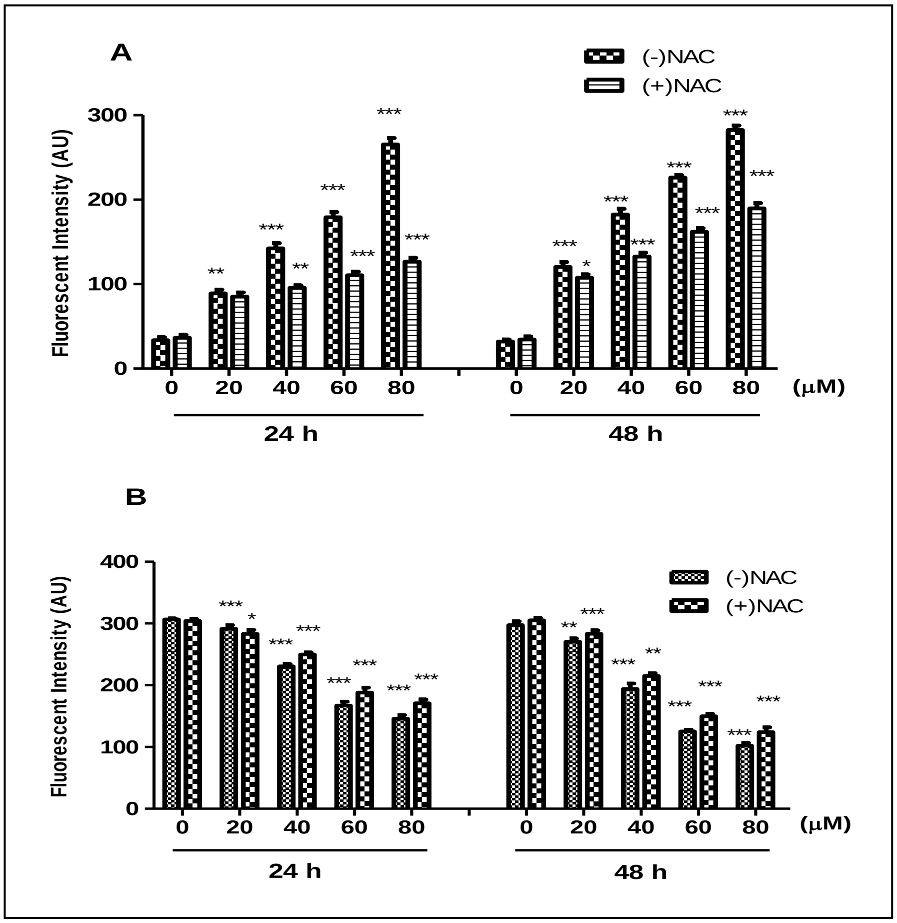

Figure S2. (A) Effect of andrographolide treatment on ROS generation in MCF-7 cells. Cells were pretreated with or without 5 mM NAC for 1 h followed by different concentrations (0, 20, 40, 60 and 80 μM) of andrographolide and incubated for the indicated times (24 h and 48 h). Intracellular ROS production was monitored by spectrofluorometer, using 2´, 7´-dichlorofluorescein diacetate (DCF-DA). (B) Loss of MMP ($\Delta\psi$m) in MCF-7 cells upon treatment with andrographolide (0-80 μM) in presence and absence of NAC. MMP was measured by spectrofluorometer using a fluorescent probe Rhodamine 123. Results shown are representative of three independent experiments. *P<0.05, **P<0.01 and ***P<0.001, when compared with control.
