# Supplementary material for: Cytotoxicity and cell cycle arrest induced by andrographolide lead to programmed cell death of MDA-MB-231 breast cancer cell line
Source: J Biomed Sci. 2016 Apr 16;23:40. doi: 10.1186/s12929-016-0257-0 (PMC4833932; doi:10.1186/s12929-016-0257-0)
Supplement: Additional file 3: — Effect of andrographolide on caspase-9 and caspase-7 activities in MCF-7 cells. Figure S3. Activation of caspase-9 (A) and caspase-7 (B) after treatment with different concentrations (0, 20, 40, 60 and 80 μM) of andrographolide in MCF-7 cells for 24 h. Results shown are representative of three independent experiments. *P < 0.05, **P < 0.01 and ***P < 0.001, when compared with control. (PDF 43 kb) [file 12929_2016_257_MOESM3_ESM.pdf]

Additional File 3

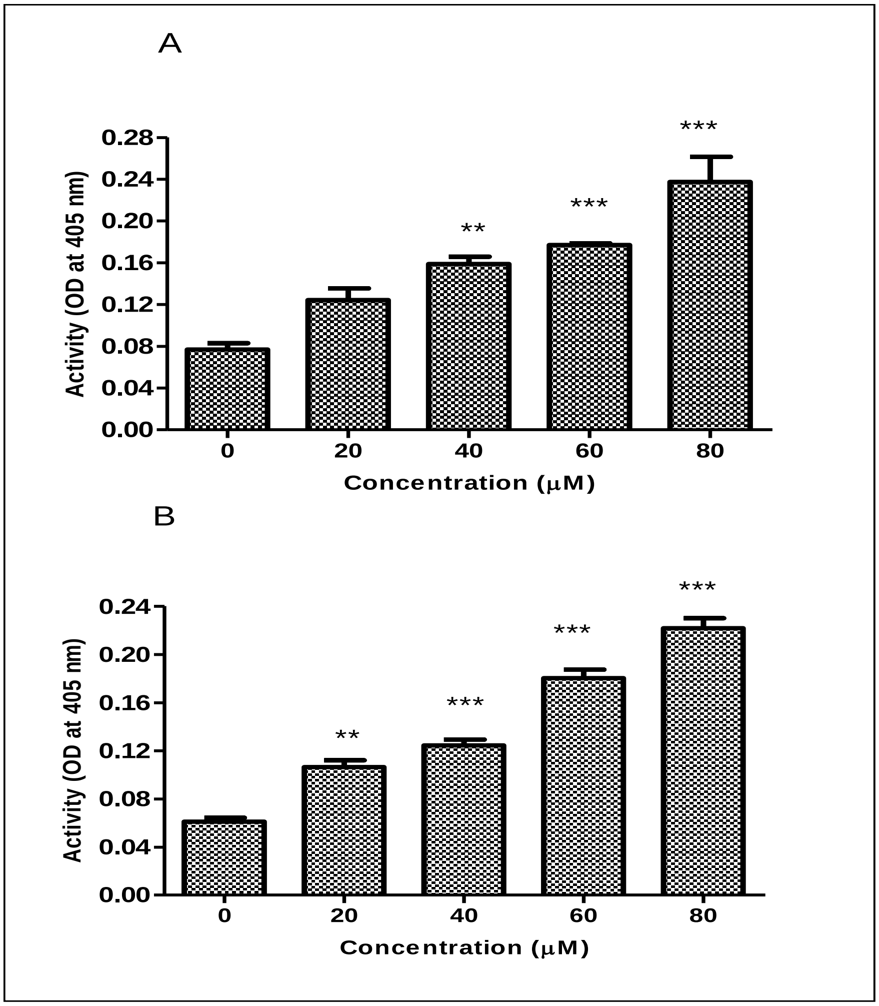

Figure S3. Activation of caspase-9 (A) and caspase-7 (B) after treatment with different concentrations (0, 20, 40, 60 and 80 μM) of andrographolide in MCF-7 cells for 24 h. Results shown are representative of three independent experiments. *P<0.05, **P<0.01 and ***P<0.001, when compared with control.
